# Supplementary material for: How Long Do Implanted Triclosan Sutures Inhibit Staphylococcus aureus in Surgical Conditions? A Pharmacological Model
Source: Pharmaceutics. 2022 Feb 28;14(3):539. doi: 10.3390/pharmaceutics14030539 (PMC8953209; doi:10.3390/pharmaceutics14030539)
Supplement: Supplementary file 1 [file pharmaceutics-14-00539-s001.zip › pharmaceutics-1568782-supplementary.pdf]

pharmaceutics

MDPI

How Long Do Implanted Triclosan Sutures Inhibit *Staphylococcus aureus* in Surgical Conditions? A Pharmacological Model

Frederic C. Daoud, Ruben Goncalves and Nicholas Moore

1. Tested Sutures:

Vicryl PLUS USP 0. Part number VCP518H. Lot QGBDRLW0. Expiry date 2023-05
Vicryl PLUS USP 1. Part number VCP359H. Lot QGBCSSW0. Expiry date 2023-05-31
Vicryl PLUS USP 2-0. Part number VCP317H. Lot QHBBHJW0. Expiry date 2023-06
PDS PLUS USP 2-0. Part number VCP317H. Lot QGMHDH. Expiry date 2022-05
Monocryl PLUS USP 2-0. Part number VCP317H. Lot QBMCT. Expiry date 2022-01
Triclosan (powder) Sigma-Aldrich PHR 1338-1G. Lot LRAC4483. Expiry date 2023-10

Table S1. In vitro water and ex vivo sample preparation schedule.

**Water without Stirring**	**Tube 1**	**Tube 2**	**Tube 3**	**Tube 4**	**Pre-Immersion**	**Marginal Immersion**
P+, M+, V+ USP 2-0 (2 samples each)	4 h	-	-	-	-	4 h
P+, M+, V+ USP 2-0 (2 samples each)	8 h	-	-	-	-	8 h
P+, M+, V+ USP 2-0 (2 samples each)	12 h	-	-	-	-	12 h
P+, M+, V+ USP 2-0 (2 samples each)	***1 d***	12 h	-	-	***1 d***	12 h
P+, M+, V+ USP 2-0 (2 samples each)	***2 d***	12 h	-	-	***2 d***	12 h
P+, M+, V+ USP 2-0 (2 samples each)	***3 d***	12 h	-	-	***3 d***	12 h
P+, M+, V+ USP 2-0 (2 samples each)	***4 d***	12 h	-	-	***4 d***	12 h
P+, M+, V+ USP 2-0 (2 samples each)	***5 d***	12 h	-	-	***5 d***	12 h
P+, M+, V+ USP 2-0 (2 samples each)	***10 d***	-	12 h	-	***10 d***	12 h
P+, M+, V+ USP 2-0 (2 samples each)	***10 d***	***5 d***	12 h	-	***15 d***	12 h
P+, M+, V+ USP 2-0 (2 samples each)	***10 d***	***10 d***	12 h	-	***20 d***	12 h
P+, M+, V+ USP 2-0 (2 samples each)	***10 d***	***10 d***	***5 d***	12 h	***25 d***	12 h
P+, M+, V+ USP 2-0 (2 samples each)	***10 d***	***10 d***	***10 d***	12 h	***30 d***	12 h
V+ USP 0 (6 samples)	***7 d***	12 h	-	-	***7 d***	12 h
V+ USP 1 (6 samples)	***7 d***	12 h	-	-	***7 d***	12 h
V+ USP 0 explanted from subcutaneous tissue (6 samples)	***7 d***	12 h	-	-	***7 d***	12 h
V+ USP 1 explanted from muscle (6 samples)	***7 d***	12 h	-	-	***7 d***	12 h

h: hours, *d*: days.

Table S2. Modeling phase–Sample preparation–EtOH/water with stirring.

**13.30% *w/w***	**13.30% *w/w***	**3.30% *w/w***	**3.30% *w/w***	**50.00% *w/w***	**50.00% *w/w***
**V+ USP 0 (2 samples)**	**V+ USP 1 (2 samples)**	**V+ USP 0 (2 samples)**	**V+ USP 1 (2 samples)**	**V+ USP 0 (2 samples)**	**V+ USP 1 (2 samples)**
1: 0 to 4 h	1: 0 to 4 h	1: 0 to 4 h	1: 0 to 4 h	1: 0 to 144 h	1: 0 to 144 h
2: 4 to 12 h	2: 4 to 12 h	2: 4 to 12 h	2: 4 to 12 h		
3: 12 to 24 h	3: 12 to 24 h	3: 12 to 24 h	3: 12 to 24 h	**50.00% *w/w***	**50.00% *w/w***
4: 24 to 36 h	4: 24 to 36 h	4: 24 to 36 h	4: 24 to 36 h	**V+ USP 2-0**	**P+ USP 2-0**

				**(2 samples)**	**(2 samples)**
5: 36 to 48 h	5: 36 to 48 h	5: 36 to 48 h	5: 36 to 48 h	1: 0 to 144 h	1: 0 to 144 h
6: 48 to 72 h	6: 48 to 72 h	6: 48 to 72 h	6: 48 to 72 h		
7: 72 to 96 h	7: 72 to 96 h	7: 72 to 96 h	7: 72 to 96 h	**50.00% *w/w***	
8: 96 to 120 h	8: 96 to 120 h	8: 96 to 120 h	8: 96 to 120 h	**M+ USP 2-0 (2 samples)**	
9: 120 to 144 h	9: 120 to 144 h	9: 120 to 144 h	9: 120 to 144 h	1: 0 to 144 h	
10: 144 to 168 h	10: 144 to 168 h	10: 144 to 168 h	10: 144 to 168 h		
11: 168 to 192 h	11: 168 to 192 h	11: 168 to 192 h	11: 168 to 192 h		

Table S3. Estimate of marginal release rate µg/m/h–in static water.

Medium	Suture	Pre-Immersion days	Immersion Hours	N	Min	Mean	Max
water	Vicryl+ 2-0	0	4	2	1.37143	1.69286	2.01429
		0	8	2	1.17143	1.17143	1.17143
		0	12	2	0.60476	0.68929	0.77381
		1	12	2	0.45238	0.61548	0.77857
		2	12	2	0.08595	0.39655	0.70714
		3	12	2	0.34762	0.63690	0.92619
		4	12	2	0.44524	0.69405	0.94286
		5	12	2	0.29762	0.75833	1.21905
		10	12	2	0.44286	0.49167	0.54048
		15	12	2	0.27381	0.27976	0.28571
		20	12	2	0.27619	0.27857	0.28095
		25	12	2	0.17333	0.19440	0.21548
		30	12	1	0.09500	0.09500	0.09500
water	PDS+ 2-0	0	4	2	2.83571	3.75357	4.67143
		0	8	2	2.11429	2.38214	2.65000
		0	12	2	1.47857	1.49048	1.50238
		1	12	2	0.79286	0.89762	1.00238
		2	12	2	0.68095	0.72619	0.77143
		3	12	2	0.71429	0.83333	0.95238
		4	12	2	0.78333	0.82857	0.87381
		5	12	2	0.72143	0.84048	0.95952
		10	12	2	0.70000	0.76786	0.83571
		15	12	2	0.64048	0.65476	0.66905
		20	12	2	0.70952	0.73571	0.76190
		25	12	2	0.60714	0.62976	0.65238
		30	12	2	0.52381	0.53333	0.54286
water	Monocryl+ 2-0	0	4	2	5.20714	5.45000	5.69286
		0	8	2	3.13929	3.43393	3.72857
		0	12	2	2.08810	2.18333	2.27857
		1	12	2	1.03810	1.24762	1.45714
		2	12	2	0.97381	1.21548	1.45714
		3	12	2	0.65952	0.99405	1.32857
		4	12	2	0.86429	1.12500	1.38571
		5	12	2	0.97619	0.99643	1.01667
		10	12	2	0.74048	0.77738	0.81429
		15	12	2	0.52143	0.59286	0.66429
		20	12	2	0.50476	0.55595	0.60714
		25	12	2	0.38810	0.41310	0.43810

30	12	2	0.49762	0.50357	0.50952	

Table S4. Ex vivo marginal release rate benchmarks versus in vitro controls (μg/m/h).

Medium/Solvent	Suture	Days Immersed	N	Min	Mean	Max	SD
Static water	V+ 0	7	6	0.17881	0.20262	0.21952	0.01388
Static water	V+ 1	7	6	0.25714	0.30397	0.34524	0.03229
subcutaneous	V+ 0	7	6	0.00913	0.01486	0.02482	0.00652
intramuscular	V+ 1	7	6	0.00345	0.00473	0.00612	0.00095

Ex Vivo Animal Benchmarking Data–Tests of Significance

Comparison of triclosan release rate of V + 0 after explantation from subcutaneous tissue on day-7 vs. immersion in pure water 7 days

ttest microgpmph if (suture == 4), unequal by(medium)

Group	Obs	Mean	Std. Err.	Std. Dev.	[95% Conf.	Interval]
Static water	6	0.2026	0.0057	0.0139	0.1881	0.2172
Subcutaneous	6	0.0149	0.0027	0.0065	0.0080	0.0217
Combined	12	0.1087	0.0285	0.0956	0.0541	0.1714
diff		0.1878	0.0062		0.2654	0.2017
diff = mean(water)-mean(subcutaneous)					t = 29.9901	
H0: diff = 0					Satterthwaite's dF = 10	
Ha: diff < 0				Ha: diff! = 0		Ha: diff > 0
Pr(T < t) = 1.0000				Pr(|T| > |t|) = 0.0000		Pr(T > t) = 0.0000

Ratio of marginal release in water/subcutaneous tissue

Mean	Coefficient	Std. Err.	z	P > z	[95% Conf.	Interval]
_nl_1	13.634	2.471	5.52	0.000	8.791	18.478

Comparison of triclosan release rate of V+1 after explantation from muscle on day-7 vs. immersion in pure water 7 days: t-test

Group	Obs	Mean	Std. Err.	Std. Dev.	[95% Conf.	Interval]
Static water	6	0.3040	0.0132	0.0323	0.2701	0.3378
muscle	6	0.0047	0.0004	0.0010	0.0037	0.0057
Combined	12	0.1544	0.0455	0.1578	0.0541	0.2546
diff		0.2992	0.0132		0.2654	0.3331
diff = mean(water)-mean(muscle)					t = 22.6931	
H0: diff = 0					Satterthwaite's dF = 5.00874	
Ha: diff < 0				Ha: diff! = 0		Ha: diff > 0
Pr(T < t) = 1.0000				Pr(|T| > |t|) = 0.0000		Pr(T > t) = 0.0000

Ratio of marginal release in water/muscle

Mean	Coefficient	Std. Err.	z	P>z	[95% Conf.	Interval]
_nl_1	64.208	5.974	10.75	0.000	52.498	75.918

Ratio of marginal release V+1 subcutaneous/V + 0 intramuscular

Mean	Coefficient	Std. Err.	z	P > z	[95% Conf.	Interval]

_nl_1	3.139	0.619	5.07	0.000	1.926	4.352

Ratio of marginal release V+1/V+0 after 7 days in water both

Mean	Coefficient	Std. Err.	z	P > z	[95% Conf.	Interval]
_nl_1	0.667	0.034	19.38	0.000	0.599	0.734

Table S5. Cumulative and marginal release rates calculated from raw data with EtOH/water solvent.

				Cumulative Release (µg/m)				Marginal Release Rate (µg/m/h)		
Solvent/Medium	Suture Type	N	Cumulative Immersion Hours	Min	Mean	Max	Marginal Immersion Hours	Min	Mean	Max
EtHO/water 3.3% *w/w*	Vicryl+ USP 0	2	4	2.685714	2.814286	2.942857	4	0.6714286	0.7035714	0.7357143
		2	12	4.928571	5.114286	5.3	12	0.2803572	0.2875	0.2946429
		2	24	6.771429	6.792857	6.814286	24	0.122619	0.139881	0.1571429
		2	36	8.342857	8.385715	8.428572	36	0.1273809	0.1327381	0.1380952
		2	48	9.274286	9.522857	9.771428	48	0.0704762	0.0947619	0.1190476
		2	72	9.951428	10.24571	10.54	72	0.0282143	0.030119	0.0320238
		2	96	10.50286	10.78143	11.06	96	0.0216667	0.0223214	0.0229762
		2	120	10.96286	11.19286	11.42286	120	0.015119	0.0171429	0.0191667
		2	144	11.45143	11.57386	11.69629	144	0.0113929	0.015875	0.0203571
		2	168	11.91657	11.92543	11.93429	168	0.0091786	0.0146488	0.020119
		2	192	12.12343	12.18171	12.24	192	0.008619	0.0106786	0.0127381
		2	216	12.27257	12.374	12.47543	216	0.0062143	0.0080119	0.0098095
EtHO/water 3.3% *w/w*	Vicryl+ USP 1	2	4	3.6	3.7	3.8	4	0.9	0.925	0.95
		2	12	6.628572	7	7.371428	12	0.3785714	0.4125	0.4464286
		2	24	9	9.435714	9.871428	24	0.197619	0.2029762	0.2083333
		2	36	10.9	11.31429	11.72857	36	0.1547619	0.1565476	0.1583333
		2	48	12.64286	12.91429	13.18571	48	0.1214286	0.1333333	0.1452381
		2	72	13.51143	13.75857	14.00571	72	0.0341667	0.0351786	0.0361905
		2	96	14.12	14.30143	14.48286	96	0.019881	0.022619	0.0253571
		2	120	14.52857	14.70429	14.88	120	0.0165476	0.0167857	0.0170238
		2	144	15.05714	15.12429	15.19143	144	0.0129762	0.0175	0.0220238
		2	168	15.34571	15.38243	15.41914	168	0.0094881	0.010756	0.0120238
		2	192	15.64571	15.74957	15.85343	192	0.0125	0.0152976	0.0180952
		2	216	15.912	16.00157	16.09114	216	0.0099048	0.0105	0.0110952
EtHO/water 13.3% *w/w*	Vicryl+ USP 0	2	4	5	5.4	5.8	4	1.25	1.35	1.45
		2	12	8.428572	8.571429	8.714286	12	0.3642857	0.3964286	0.4285714
		2	24	10.52857	10.67143	10.81429	24	0.175	0.175	0.175
		2	36	12.38571	12.52857	12.67143	36	0.1547619	0.1547619	0.1547619
		2	48	13.61143	14.25143	14.89143	48	0.1021429	0.1435714	0.185
		2	72	14.48	14.97286	15.46571	72	0.0239286	0.0300595	0.0361905
		2	96	15.29714	15.71572	16.13429	96	0.0278571	0.0309524	0.0340476
		2	120	15.95429	16.29	16.62572	120	0.0204762	0.0239286	0.027381
		2	144	16.58571	16.85143	17.11714	144	0.0204762	0.0233929	0.0263095
		2	168	16.97714	17.21286	17.44857	168	0.0138095	0.0150595	0.0163095
		2	192	17.22486	17.44243	17.66	192	0.0088095	0.0095655	0.0103214
EtHO/water 13.3% *w/w*	Vicryl+ USP 1	2	4	6.8	6.871429	6.942857	4	1.7	1.717857	1.735714
		2	12	12.68571	13.05714	13.42857	12	0.7357143	0.7732143	0.8107143
		2	24	16.17143	16.42857	16.68571	24	0.2714286	0.2809524	0.2904762
		2	36	18.2	18.27143	18.34286	36	0.1380952	0.1535714	0.1690476
		2	48	19.39429	20.01	20.62572	48	0.087619	0.144881	0.2021429
		2	72	20.03429	20.72	21.40571	72	0.0266667	0.0295833	0.0325
		2	96	20.59143	21.29714	22.00286	96	0.0232143	0.0240476	0.024881

	2	120	20.92571	21.71286	22.5	120	0.0139286	0.0173214	0.0207143
	2	144	21.26	22.08143	22.90286	144	0.0139286	0.0153571	0.0167857
	2	168	21.43714	22.39286	23.34857	168	0.007381	0.0129762	0.0185714
	2	192	21.77143	22.65743	23.54343	192	0.008119	0.0110238	0.0139286

Table S6. Residual amount of triclosan over time with in EtOH/water 13.3% *w*/*w*.

Suture/Method	A0	4 h	12 h	24 h	48 h	72 h	96 h	120 h	144 h	168 h	174 h
V+ 0 measured	48.1	42.7	39.5	37.4	33.8	33.1	32.4	31.8	31.2	30.9	30.8
V+ 0 predicted	48.1	42	39.3	37.0	33.9	32.1	31.1	30.4	30.1	29.8	29.8
V+ 1 measured	52.3	45.4	39.2	35.9	32.3	31.6	31.0	30.6	30.2	29.9	29.8
V+ 1 predicted	52.3	45.1	40.1	36.7	33.1	31.6	30.9	30.6	30.5	30.4	30.4

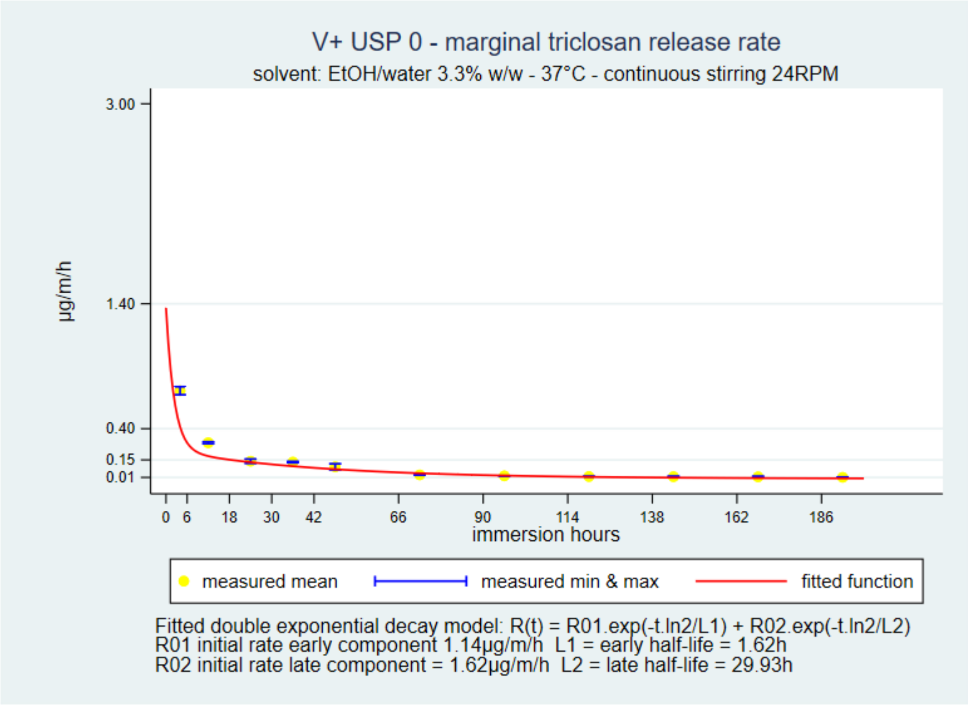

Figure S1. V+ USP 0 in 3.3% *w*/*w* release rates and double exponential fitting.

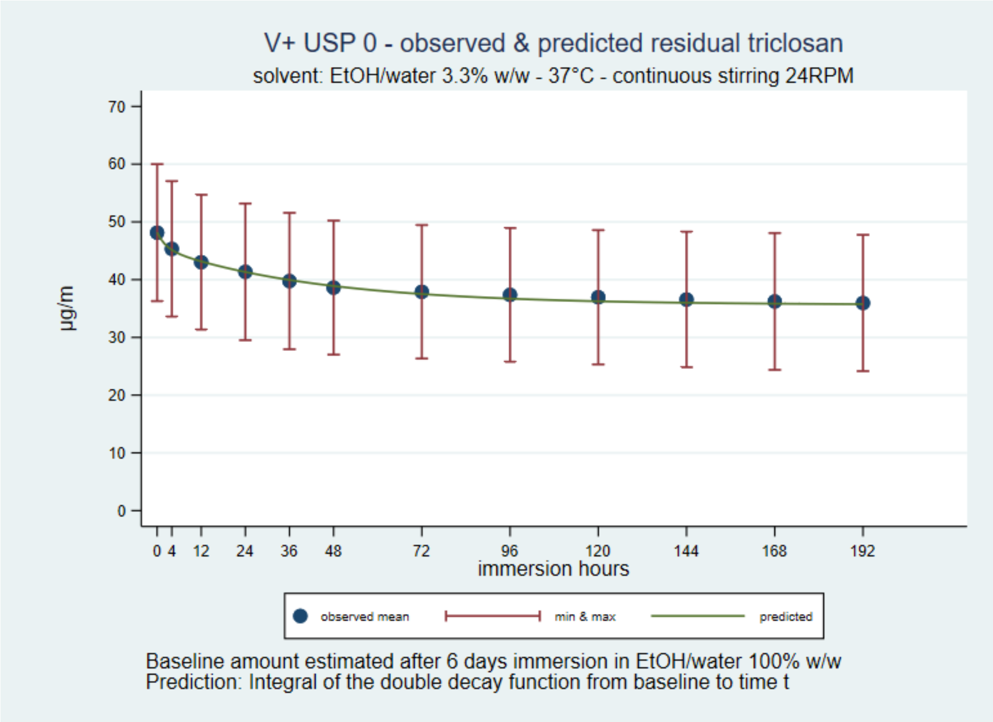

Figure S2. V+ USP 0 in 3.3% *w/w* predicted residual triclosan.

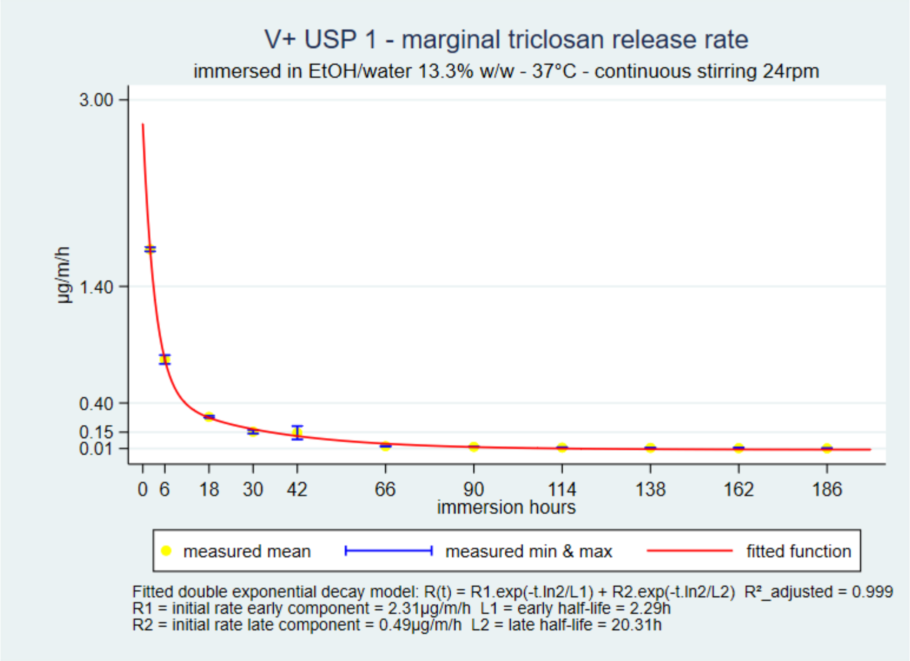

Figure S3. V+ USP 1 in 13.3% *w/w* release rates and double exponential fitting.

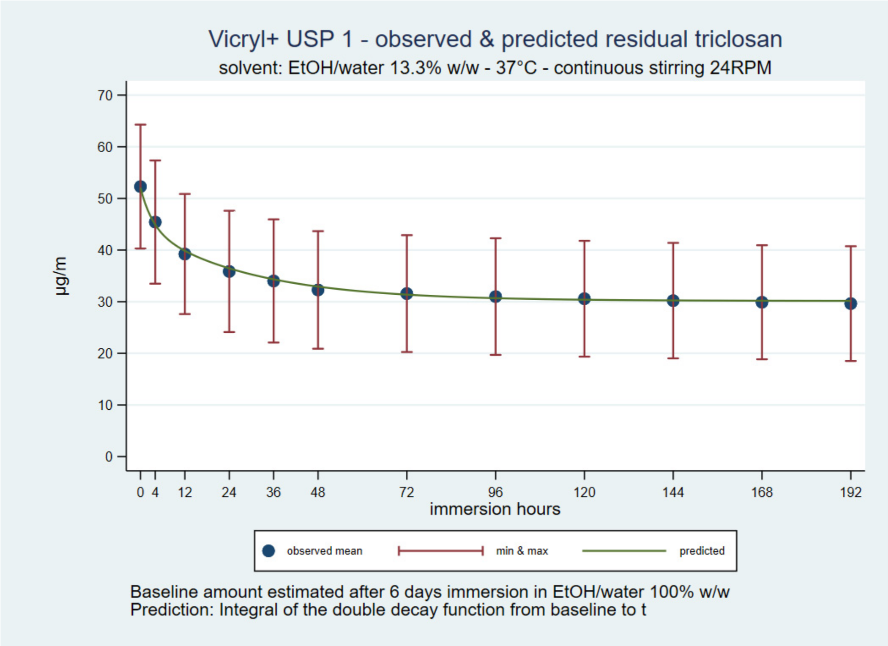

Figure S4. V+ USP 1 in 13.3% *w*/*w* predicted residual triclosan.

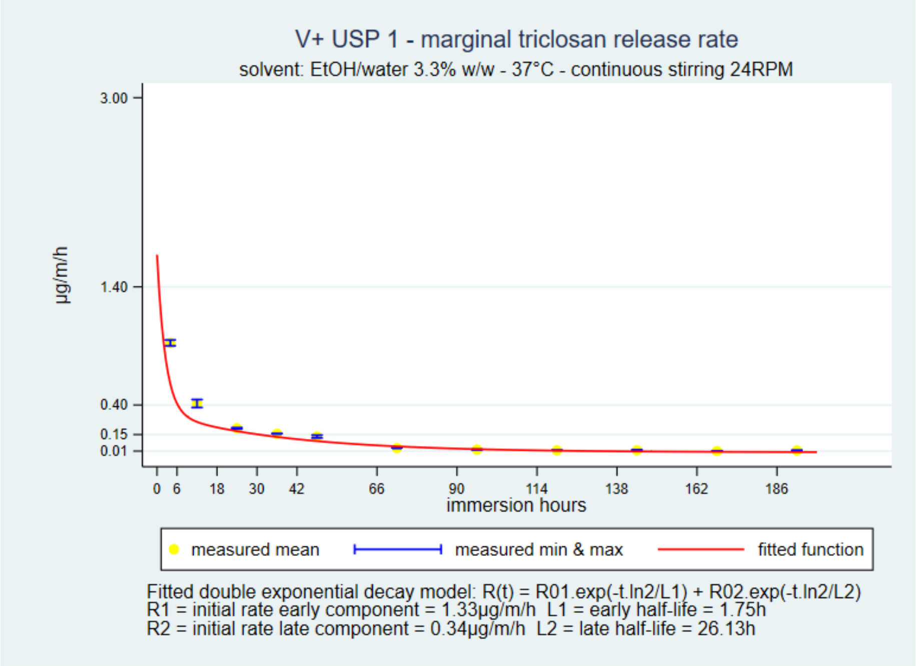

Figure S5. V+ USP 1 in 3.3% *w*/*w* release rates and double exponential fitting.

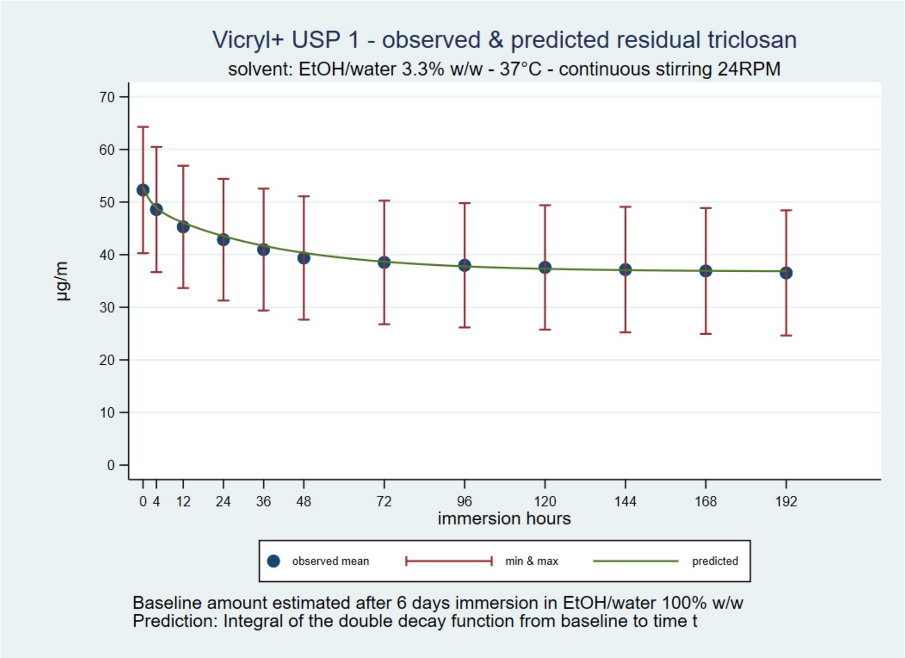

Figure S6. V+ USP 1 in 3.3% *w/w* predicted residual triclosan.

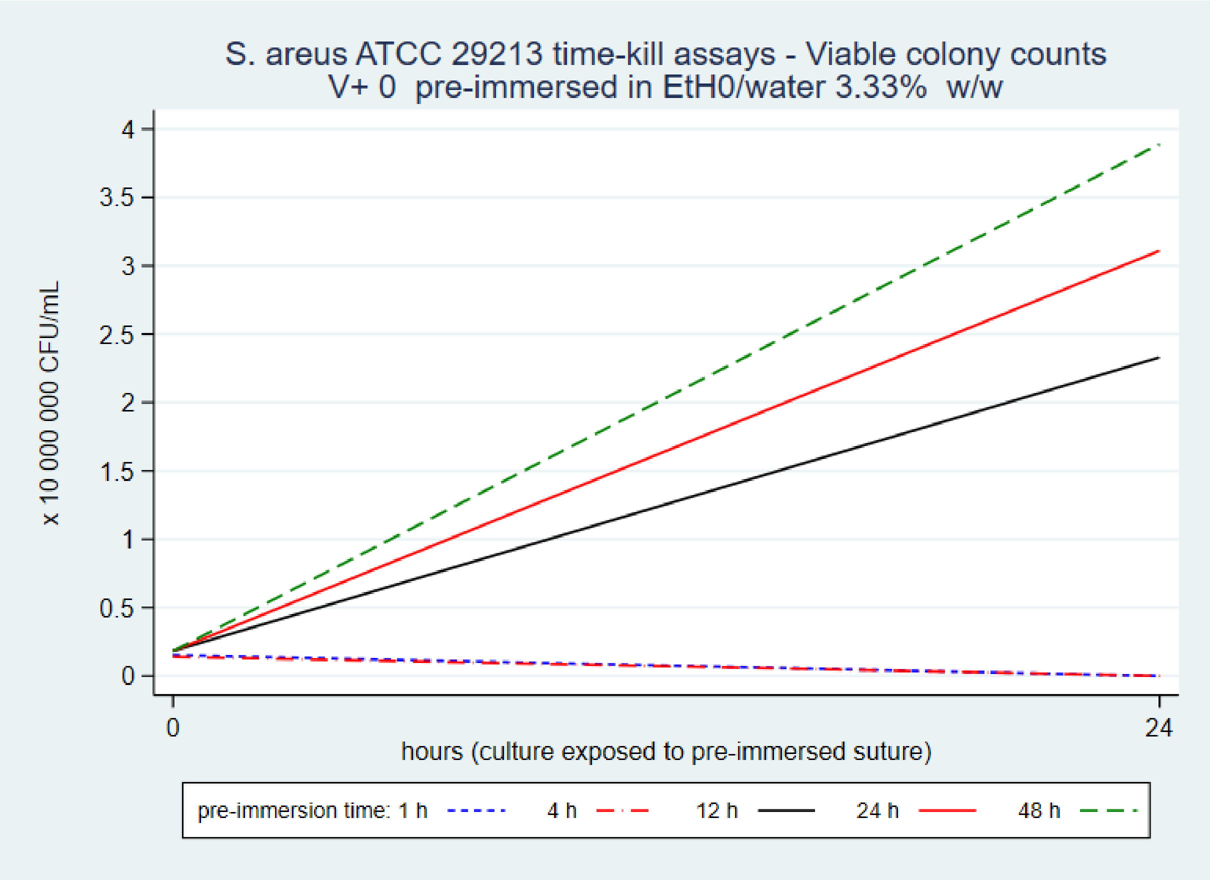

Figure S7. V+ USP 0 in 3.3% *w/w* time-kill assay.

Raw Data

Raw data-Exploratory phase: Static water immersion & animal explanted sutures

Sample Id	Tube	Suture Type & Caliber (USP)	Solvent/Medium	Solvent Volume (L)	Segment Length (m)	Immersion Time to Determination (hours)	Pre-Immmersion Time (Hours)	Triclosan Concentration (μg/L)
19822	34 C Neg	control	water	0.01	0.35	0	0	0
19823	1 V+	Vicryl+ 2-0	water	0.01	0.35	4	0	192
19824	2 V+	Vicryl+ 2-0	water	0.01	0.35	4	0	282
19825	7 V+	Vicryl+ 2-0	water	0.01	0.35	8	0	328
19826	8 V+	Vicryl+ 2-0	water	0.01	0.35	8	0	328
19827	13 V+	Vicryl+ 2-0	water	0.01	0.35	12	0	325
19828	14 V+	Vicryl+ 2-0	water	0.01	0.35	12	0	254
19829	19B V+	Vicryl+ 2-0	water	0.01	0.35	12	24	327
19830	22B V+	Vicryl+ 2-0	water	0.01	0.35	12	48	297
19831	25B V+	Vicryl+ 2-0	water	0.01	0.35	12	72	389
19832	28B V+	Vicryl+ 2-0	water	0.01	0.35	12	96	396
19833	31B V+	Vicryl+ 2-0	water	0.01	0.35	12	120	512
19834	19A V+	Vicryl+ 2-0	water	0.01	0.35	24	0	676
19835	22A V+	Vicryl+ 2-0	water	0.01	0.35	48	0	612
19836	25A V+	Vicryl+ 2-0	water	0.01	0.35	72	0	900
19837	28A V+	Vicryl+ 2-0	water	0.01	0.35	96	0	672
19838	31A V+	Vicryl+ 2-0	water	0.01	0.35	120	0	776
19839	3 P+	PDS+ 2-0	water	0.01	0.35	4	0	397
19840	4 P+	PDS+ 2-0	water	0.01	0.35	4	0	654
19841	9 P+	PDS+ 2-0	water	0.01	0.35	8	0	742
19842	10 P+	PDS+ 2-0	water	0.01	0.35	8	0	592
19843	15 P+	PDS+ 2-0	water	0.01	0.35	12	0	631
19844	16 P+	PDS+ 2-0	water	0.01	0.35	12	0	621
19845	20B P+	PDS+ 2-0	water	0.01	0.35	12	24	421
19846	23B P+	PDS+ 2-0	water	0.01	0.35	12	48	324
19847	26B P+	PDS+ 2-0	water	0.01	0.35	12	72	400
19848	29B P+	PDS+ 2-0	water	0.01	0.35	12	96	367
19849	32B P+	PDS+ 2-0	water	0.01	0.35	12	120	403
19850	20A P+	PDS+ 2-0	water	0.01	0.35	24	0	608
19851	23A P+	PDS+ 2-0	water	0.01	0.35	48	0	575
19852	26A P+	PDS+ 2-0	water	0.01	0.35	72	0	465
19853	29A P+	PDS+ 2-0	water	0.01	0.35	96	0	507
19854	32A P+	PDS+ 2-0	water	0.01	0.35	120	0	474
19855	5 M+	Monocryl+ 2-0	water	0.01	0.35	4	0	797
19856	6 M+	Monocryl+ 2-0	water	0.01	0.35	4	0	729
19857	11 M+	Monocryl+ 2-0	water	0.01	0.35	8	0	879
19858	12 M+	Monocryl+ 2-0	water	0.01	0.35	8	0	1044
19859	17 M+	Monocryl+ 2-0	water	0.01	0.35	12	0	957
19860	18 M+	Monocryl+ 2-0	water	0.01	0.35	12	0	877
19861	21B M+	Monocryl+ 2-0	water	0.01	0.35	12	24	612
19862	24B M+	Monocryl+ 2-0	water	0.01	0.35	12	48	612
19863	27B M+	Monocryl+ 2-0	water	0.01	0.35	12	72	558
19864	30B M+	Monocryl+ 2-0	water	0.01	0.35	12	96	582
19865	33B M+	Monocryl+ 2-0	water	0.01	0.35	12	120	427
19866	21A M+	Monocryl+ 2-0	water	0.01	0.35	24	0	741
19867	24A M+	Monocryl+ 2-0	water	0.01	0.35	48	0	776
19868	27A M+	Monocryl+ 2-0	water	0.01	0.35	72	0	571
19869	30A M+	Monocryl+ 2-0	water	0.01	0.35	96	0	569
19870	33A M+	Monocryl+ 2-0	water	0.01	0.35	120	0	571

1322	150B NEGATIVE CON	control	water	0.01	0.35	0	0	0
1335	134B V+	Vicryl+ 2-0	water	0.01	0.35	12	24	190
1336	131B V+	Vicryl+ 2-0	water	0.01	0.35	12	48	36.1
1337	128B V+	Vicryl+ 2-0	water	0.01	0.35	12	72	146
1338	125B V+	Vicryl+ 2-0	water	0.01	0.35	12	96	187
1339	122B V+	Vicryl+ 2-0	water	0.01	0.35	12	120	125
1340	119B2 V+	Vicryl+ 2-0	water	0.01	0.35	12	240	186
1341	119B1 V+	Vicryl+ 2-0	water	0.01	0.35	12	240	227
1342	122B2 V+	Vicryl+ 2-0	water	0.01	0.35	12	360	120
1343	122B1 V+	Vicryl+ 2-0	water	0.01	0.35	12	360	115
1344	125B2 V+	Vicryl+ 2-0	water	0.01	0.35	12	480	118
1345	125B1 V+	Vicryl+ 2-0	water	0.01	0.35	12	480	116
1346	128B2 V+	Vicryl+ 2-0	water	0.01	0.35	12	600	72.8
1347	128B1 V+	Vicryl+ 2-0	water	0.01	0.35	12	600	90.5
1348	131B2 V+	Vicryl+ 2-0	water	0.01	0.35	12	720	39.9
1350	135B P+	PDS+ 2-0	water	0.01	0.35	12	24	333
1351	132B P+	PDS+ 2-0	water	0.01	0.35	12	48	286
1352	129B P+	PDS+ 2-0	water	0.01	0.35	12	72	300
1353	126B P+	PDS+ 2-0	water	0.01	0.35	12	96	329
1354	123B P+	PDS+ 2-0	water	0.01	0.35	12	120	303
1355	120B2 P+	PDS+ 2-0	water	0.01	0.35	12	240	351
1356	120B1 P+	PDS+ 2-0	water	0.01	0.35	12	240	294
1357	123B2 P+	PDS+ 2-0	water	0.01	0.35	12	360	269
1358	123B1 P+	PDS+ 2-0	water	0.01	0.35	12	360	281
1359	126B2 P+	PDS+ 2-0	water	0.01	0.35	12	480	320
1360	126B1 P+	PDS+ 2-0	water	0.01	0.35	12	480	298
1361	129B2 P+	PDS+ 2-0	water	0.01	0.35	12	600	255
1362	129B1 P+	PDS+ 2-0	water	0.01	0.35	12	600	274
1363	132B2 P+	PDS+ 2-0	water	0.01	0.35	12	720	228
1364	132B1 P+	PDS+ 2-0	water	0.01	0.35	12	720	220
1365	136B M+	Monocryl+ 2-0	water	0.01	0.35	12	24	436
1366	133B M+	Monocryl+ 2-0	water	0.01	0.35	12	48	409
1367	130B M+	Monocryl+ 2-0	water	0.01	0.35	12	72	277
1368	127B M+	Monocryl+ 2-0	water	0.01	0.35	12	96	363
1369	124B M+	Monocryl+ 2-0	water	0.01	0.35	12	120	410
1370	121B2 M+	Monocryl+ 2-0	water	0.01	0.35	12	240	311
1371	121B1 M+	Monocryl+ 2-0	water	0.01	0.35	12	240	342
1372	124B2 M+	Monocryl+ 2-0	water	0.01	0.35	12	360	279
1373	124B1 M+	Monocryl+ 2-0	water	0.01	0.35	12	360	219
1374	127B2 M+	Monocryl+ 2-0	water	0.01	0.35	12	480	255
1375	127B1 M+	Monocryl+ 2-0	water	0.01	0.35	12	480	212
1376	130B2 M+	Monocryl+ 2-0	water	0.01	0.35	12	600	163
1377	130B1 M+	Monocryl+ 2-0	water	0.01	0.35	12	600	184
1378	133B2 M+	Monocryl+ 2-0	water	0.01	0.35	12	720	209
1379	133B1 M+	Monocryl+ 2-0	water	0.01	0.35	12	720	214
1310	V-0-C1.1-B	Vicryl+ 0	subcutaneous	0.0063	0.22	12	168	4.11
1311	V-0-C1.2-B	Vicryl+ 0	subcutaneous	0.0063	0.22	12	168	3.97
1312	V-0-C2.1-B	Vicryl+ 0	subcutaneous	0.0063	0.22	12	168	6.76
1313	V-0-C2.2-B	Vicryl+ 0	subcutaneous	0.0063	0.22	12	168	10.4
1318	V-0-C3.1-B	Vicryl+ 0	subcutaneous	0.00495	0.1725	12	168	3.82
1319	V-0-C3.2-B	Vicryl+ 0	subcutaneous	0.00495	0.1725	12	168	8.28
1314	V-1-M1.1-B	Vicryl+ 1	intramuscular	0.01	0.35	12	168	1.45
1315	V-1-M1.2-B	Vicryl+ 1	intramuscular	0.01	0.35	12	168	2.57
1316	V-1-M2.1-B	Vicryl+ 1	intramuscular	0.01	0.35	12	168	2.04
1317	V-1-M2.2-B	Vicryl+ 1	intramuscular	0.01	0.35	12	168	2.13
1320	V-1-M3.1-B	Vicryl+ 1	intramuscular	0.01	0.35	12	168	1.62
1321	V-1-M3.2-B	Vicryl+ 1	intramuscular	0.01	0.35	12	168	2.12
1323	V-0-C1-B	Vicryl+ 0	water	0.01	0.35	12	168	82.5
1324	V-0-C2-B	Vicryl+ 0	water	0.01	0.35	12	168	75.1

1325	V-0-C3-B	Vicryl+ 0	water	0.01	0.35	12	168	87.1
1326	V-0-C4-B	Vicryl+ 0	water	0.01	0.35	12	168	85.7
1327	V-0-C5-B	Vicryl+ 0	water	0.01	0.35	12	168	88
1328	V-0-C6-B	Vicryl+ 0	water	0.01	0.35	12	168	92.2
1329	V-1-C1-B	Vicryl+ 1	water	0.01	0.35	12	168	145
1330	V-1-C2-B	Vicryl+ 1	water	0.01	0.35	12	168	141
1331	V-1-C3-B	Vicryl+ 1	water	0.01	0.35	12	168	127
1332	V-1-C4-B	Vicryl+ 1	water	0.01	0.35	12	168	122
1333	V-1-C5-B	Vicryl+ 1	water	0.01	0.35	12	168	108
1334	V-1-C6-B	Vicryl+ 1	water	0.01	0.35	12	168	123
8925	V0.p.total.1	Vicryl+ 0	EtHO/H20 100% *w/w*	0.01	0.35		144	2100
8926	V1.p.total.1	Vicryl+ 1	EtHO/H20 100% *w/w*	0.01	0.35		144	2250
8927	V2-0.p.total.1	Vicryl+ 2-0	EtHO/H20 100% *w/w*	0.01	0.35		144	1860
8928	PDS2-0.p.total.1	PDS+ 2-0	EtHO/H20 100% *w/w*	0.01	0.35		144	16500
8929	V0.p.total.2	Vicryl+ 0	EtHO/H20 100% *w/w*	0.01	0.35		144	1270
8930	V1.p.total.2	Vicryl+ 1	EtHO/H20 100% *w/w*	0.01	0.35		144	1410
8931	V2-0.p.total.2	Vicryl+ 2-0	EtHO/H20 100% *w/w*	0.01	0.35		144	1470
8932	PDS2-0.p.total.2	PDS+ 2-0	EtHO/H20 100% *w/w*	0.01	0.35		144	22100
8933	M2-0.p.total.1	Monocryl+ 2-0	EtHO/H20 100% *w/w*	0.01	0.35		144	41000
8934	M2-0.p.total.2	Monocryl+ 2-0	EtHO/H20 100% *w/w*	0.01	0.35		144	34000

Raw data-Modeling phase: Ethanol/Water 13.3% *w/w* and 3.3% *w/w* solutions

Sample Id	Tube	Suture Type & Caliber (USP)	Solvent/Medium	Solvent Volume (L)	Segment Length (m)	Immersion Time to Determination (Hours)	Triclosan Concentration Measured (μg/L)
8833	V0p.1.33_0_4	Vicryl+ 0	EtHO/H20 3.3% *w/w*	0.01	0.35	4	103
8834	V0p.2.33_0_4	Vicryl+ 0	EtHO/H20 3.3% *w/w*	0.01	0.35	4	94
8837	V0p.1.33_4_12	Vicryl+ 0	EtHO/H20 3.3% *w/w*	0.01	0.35	8	82.5
8838	V0p.2.33_4_12	Vicryl+ 0	EtHO/H20 3.3% *w/w*	0.01	0.35	8	78.5
8841	V0p.1.33_12_24	Vicryl+ 0	EtHO/H20 3.3% *w/w*	0.01	0.35	12	51.5
8842	V0p.2.33_12_24	Vicryl+ 0	EtHO/H20 3.3% *w/w*	0.01	0.35	12	66
8845	V0p.1.33_24_36	Vicryl+ 0	EtHO/H20 3.3% *w/w*	0.01	0.35	12	58
8846	V0p.2.33_24_36	Vicryl+ 0	EtHO/H20 3.3% *w/w*	0.01	0.35	12	53.5
8849	V0p.1.33_36_48	Vicryl+ 0	EtHO/H20 3.3% *w/w*	0.01	0.35	12	29.6
8850	V0p.2.33_36_48	Vicryl+ 0	EtHO/H20 3.3% *w/w*	0.01	0.35	12	50
8853	V0p.1.33_48_72	Vicryl+ 0	EtHO/H20 3.3% *w/w*	0.01	0.35	24	23.7
8854	V0p.2.33_48_72	Vicryl+ 0	EtHO/H20 3.3% *w/w*	0.01	0.35	24	26.9
8857	V0p.1.33_72_96	Vicryl+ 0	EtHO/H20 3.3% *w/w*	0.01	0.35	24	19.3
8858	V0p.2.33_72_96	Vicryl+ 0	EtHO/H20 3.3% *w/w*	0.01	0.35	24	18.2
8861	V0p.1.33_96_120	Vicryl+ 0	EtHO/H20 3.3% *w/w*	0.01	0.35	24	16.1
8862	V0p.2.33_96_120	Vicryl+ 0	EtHO/H20 3.3% *w/w*	0.01	0.35	24	12.7
8865	V0p.1.33_120_144	Vicryl+ 0	EtHO/H20 3.3% *w/w*	0.01	0.35	24	17.1
8866	V0p.2.33_120_144	Vicryl+ 0	EtHO/H20 3.3% *w/w*	0.01	0.35	24	9.57
8869	V0p.1.33_144_168	Vicryl+ 0	EtHO/H20 3.3% *w/w*	0.01	0.35	24	16.9
8870	V0p.2.33_144_168	Vicryl+ 0	EtHO/H20 3.3% *w/w*	0.01	0.35	24	7.71
8873	V0p.1.33_168_192	Vicryl+ 0	EtHO/H20 3.3% *w/w*	0.01	0.35	24	10.7
8874	V0p.2.33_168_192	Vicryl+ 0	EtHO/H20 3.3% *w/w*	0.01	0.35	24	7.24
8877	V0p.1.33_192_216	Vicryl+ 0	EtHO/H20 3.3% *w/w*	0.01	0.35	24	8.24
8878	V0p.2.33_192_216	Vicryl+ 0	EtHO/H20 3.3% *w/w*	0.01	0.35	24	5.22
8835	V1p.1.33_0_4	Vicryl+ 1	EtHO/H20 3.3% *w/w*	0.01	0.35	4	133

8836	V1p.2.33_0_4	Vicryl+ 1	EtHO/H20 3.3% *w/w*	0.01	0.35	4	126
8839	V1p.1.33_4_12	Vicryl+ 1	EtHO/H20 3.3% *w/w*	0.01	0.35	8	125
8840	V1p.2.33_4_12	Vicryl+ 1	EtHO/H20 3.3% *w/w*	0.01	0.35	8	106
8843	V1p.1.33_12_24	Vicryl+ 1	EtHO/H20 3.3% *w/w*	0.01	0.35	12	87.5
8844	V1p.2.33_12_24	Vicryl+ 1	EtHO/H20 3.3% *w/w*	0.01	0.35	12	83
8847	V1p.1.33_24_36	Vicryl+ 1	EtHO/H20 3.3% *w/w*	0.01	0.35	12	65
8848	V1p.2.33_24_36	Vicryl+ 1	EtHO/H20 3.3% *w/w*	0.01	0.35	12	66.5
8851	V1p.1.33_36_48	Vicryl+ 1	EtHO/H20 3.3% *w/w*	0.01	0.35	12	51
8852	V1p.2.33_36_48	Vicryl+ 1	EtHO/H20 3.3% *w/w*	0.01	0.35	12	61
8855	V1p.1.33_48_72	Vicryl+ 1	EtHO/H20 3.3% *w/w*	0.01	0.35	24	28.7
8856	V1p.2.33_48_72	Vicryl+ 1	EtHO/H20 3.3% *w/w*	0.01	0.35	24	30.4
8859	V1p.1.33_72_96	Vicryl+ 1	EtHO/H20 3.3% *w/w*	0.01	0.35	24	16.7
8860	V1p.2.33_72_96	Vicryl+ 1	EtHO/H20 3.3% *w/w*	0.01	0.35	24	21.3
8863	V1p.1.33_96_120	Vicryl+ 1	EtHO/H20 3.3% *w/w*	0.01	0.35	24	13.9
8864	V1p.2.33_96_120	Vicryl+ 1	EtHO/H20 3.3% *w/w*	0.01	0.35	24	14.3
8867	V1p.1.33_120_144	Vicryl+ 1	EtHO/H20 3.3% *w/w*	0.01	0.35	24	10.9
8868	V1p.2.33_120_144	Vicryl+ 1	EtHO/H20 3.3% *w/w*	0.01	0.35	24	18.5
8871	V1p.1.33_144_168	Vicryl+ 1	EtHO/H20 3.3% *w/w*	0.01	0.35	24	7.97
8872	V1p.2.33_144_168	Vicryl+ 1	EtHO/H20 3.3% *w/w*	0.01	0.35	24	10.1
8875	V1p.1.33_168_192	Vicryl+ 1	EtHO/H20 3.3% *w/w*	0.01	0.35	24	15.2
8876	V1p.2.33_168_192	Vicryl+ 1	EtHO/H20 3.3% *w/w*	0.01	0.35	24	10.5
8879	V1p.1.33_192_216	Vicryl+ 1	EtHO/H20 3.3% *w/w*	0.01	0.35	24	8.32
8880	V1p.2.33_192_216	Vicryl+ 1	EtHO/H20 3.3% *w/w*	0.01	0.35	24	9.32
8881	V0p.1.133_0_4	Vicryl+ 0	EtHO/H20 13.3% *w/w*	0.01	0.35	4	203
8882	V0p.2.133_0_4	Vicryl+ 0	EtHO/H20 13.3% *w/w*	0.01	0.35	4	175
8885	V0p.1.133_4_12	Vicryl+ 0	EtHO/H20 13.3% *w/w*	0.01	0.35	8	102
8886	V0p.2.133_4_12	Vicryl+ 0	EtHO/H20 13.3% *w/w*	0.01	0.35	8	120
8889	V0p.1.133_12_24	Vicryl+ 0	EtHO/H20 13.3% *w/w*	0.01	0.35	12	73.5
8890	V0p.2.133_12_24	Vicryl+ 0	EtHO/H20 13.3% *w/w*	0.01	0.35	12	73.5
8893	V0p.1.133_24_36	Vicryl+ 0	EtHO/H20 13.3% *w/w*	0.01	0.35	12	65
8894	V0p.2.133_24_36	Vicryl+ 0	EtHO/H20 13.3% *w/w*	0.01	0.35	12	65
8897	V0p.1.133_36_48	Vicryl+ 0	EtHO/H20 13.3% *w/w*	0.01	0.35	12	77.7
8898	V0p.2.133_36_48	Vicryl+ 0	EtHO/H20 13.3% *w/w*	0.01	0.35	12	42.9
8901	V0p.1.133_48_72	Vicryl+ 0	EtHO/H20 13.3% *w/w*	0.01	0.35	24	20.1
8902	V0p.2.133_48_72	Vicryl+ 0	EtHO/H20 13.3% *w/w*	0.01	0.35	24	30.4
8905	V0p.1.133_72_96	Vicryl+ 0	EtHO/H20 13.3% *w/w*	0.01	0.35	24	23.4
8906	V0p.2.133_72_96	Vicryl+ 0	EtHO/H20 13.3% *w/w*	0.01	0.35	24	28.6
8909	V0p.1.133_96_120	Vicryl+ 0	EtHO/H20 13.3% *w/w*	0.01	0.35	24	17.2
8910	V0p.2.133_96_120	Vicryl+ 0	EtHO/H20 13.3% *w/w*	0.01	0.35	24	23
8913	V0p.1.133_120_144	Vicryl+ 0	EtHO/H20 13.3% *w/w*	0.01	0.35	24	17.2
8914	V0p.2.133_120_144	Vicryl+ 0	EtHO/H20 13.3% *w/w*	0.01	0.35	24	22.1
8917	V0p.1.133_144_168	Vicryl+ 0	EtHO/H20 13.3% *w/w*	0.01	0.35	24	11.6
8918	V0p.2.133_144_168	Vicryl+ 0	EtHO/H20 13.3% *w/w*	0.01	0.35	24	13.7
8921	V0p.1.133_168_192	Vicryl+ 0	EtHO/H20 13.3% *w/w*	0.01	0.35	24	7.4
8922	V0p.2.133_168_192	Vicryl+ 0	EtHO/H20 13.3% *w/w*	0.01	0.35	24	8.67
8883	V1p.1.133_0_4	Vicryl+ 1	EtHO/H20 13.3% *w/w*	0.01	0.35	4	243
8884	V1p.2.133_0_4	Vicryl+ 1	EtHO/H20 13.3% *w/w*	0.01	0.35	4	238
8887	V1p.1.133_4_12	Vicryl+ 1	EtHO/H20 13.3% *w/w*	0.01	0.35	8	227
8888	V1p.2.133_4_12	Vicryl+ 1	EtHO/H20 13.3% *w/w*	0.01	0.35	8	206
8891	V1p.1.133_12_24	Vicryl+ 1	EtHO/H20 13.3% *w/w*	0.01	0.35	12	114
8892	V1p.2.133_12_24	Vicryl+ 1	EtHO/H20 13.3% *w/w*	0.01	0.35	12	122
8895	V1p.1.133_24_36	Vicryl+ 1	EtHO/H20 13.3% *w/w*	0.01	0.35	12	58
8896	V1p.2.133_24_36	Vicryl+ 1	EtHO/H20 13.3% *w/w*	0.01	0.35	12	71
8899	V1p.1.133_36_48	Vicryl+ 1	EtHO/H20 13.3% *w/w*	0.01	0.35	12	36.8
8900	V1p.2.133_36_48	Vicryl+ 1	EtHO/H20 13.3% *w/w*	0.01	0.35	12	84.9
8903	V1p.1.133_48_72	Vicryl+ 1	EtHO/H20 13.3% *w/w*	0.01	0.35	24	22.4
8904	V1p.2.133_48_72	Vicryl+ 1	EtHO/H20 13.3% *w/w*	0.01	0.35	24	27.3
8907	V1p.1.133_72_96	Vicryl+ 1	EtHO/H20 13.3% *w/w*	0.01	0.35	24	19.5
8908	V1p.2.133_72_96	Vicryl+ 1	EtHO/H20 13.3% *w/w*	0.01	0.35	24	20.9
8911	V1p.1.133_96_120	Vicryl+ 1	EtHO/H20 13.3% *w/w*	0.01	0.35	24	11.7
8912	V1p.2.133_96_120	Vicryl+ 1	EtHO/H20 13.3% *w/w*	0.01	0.35	24	17.4
8915	V1p.1.133_120_144	Vicryl+ 1	EtHO/H20 13.3% *w/w*	0.01	0.35	24	11.7
8916	V1p.2.133_120_144	Vicryl+ 1	EtHO/H20 13.3% *w/w*	0.01	0.35	24	14.1

8919	V1p.1.133_144_168	Vicryl+ 1	EtHO/H20 13.3% *w/w*	0.01	0.35	24	6.2
8920	V1p.2.133_144_168	Vicryl+ 1	EtHO/H20 13.3% *w/w*	0.01	0.35	24	15.6
8923	V1p.1.133_168_192	Vicryl+ 1	EtHO/H20 13.3% *w/w*	0.01	0.35	24	11.7
8924	V1p.2.133_168_192	Vicryl+ 1	EtHO/H20 13.3% *w/w*	0.01	0.35	24	6.82
8927	V2-0p. Total1	Vicryl+ 2-0	EtHO/H20 100% *w/w*	0.01	0.35	144	1860
8931	V2-0p. Total2	Vicryl+ 2-0	EtHO/H20 100% *w/w*	0.01	0.35	144	1470
8928	PDS2-0p. Total1	PDS+ 2-0	EtHO/H20 100% *w/w*	0.01	0.35	144	16500
8932	PDS2-0p. Total2	PDS+ 2-0	EtHO/H20 100% *w/w*	0.01	0.35	144	22100
8933	M2-0p. Total1	Monocryl+ 2-0	EtHO/H20 100% *w/w*	0.01	0.35	144	41000
8934	M2-0p. Total2	Monocryl+ 2-0	EtHO/H20 100% *w/w*	0.01	0.35	144	34000
8925	V0p.Total1	Vicryl+ 0	EtHO/H20 100% *w/w*	0.01	0.35	144	2100
8929	V0p.Total2	Vicryl+ 0	EtHO/H20 100% *w/w*	0.01	0.35	144	1270
8926	V1p.Total1	Vicryl+ 1	EtHO/H20 100% *w/w*	0.01	0.35	144	2250
8930	V1p.Total2	Vicryl+ 1	EtHO/H20 100% *w/w*	0.01	0.35	144	1410

Raw data–24-h Time-kill analysis *S. aureus* ATCC 29213

Solvent	Immersion Hours	Hour of Count	Count
1	1	0	1,640,000
1	4	0	1,640,000
1	12	0	1,810,000
1	24	0	1,760,000
1	48	0	2,400,000
1	1	24	0
1	4	24	0
1	12	24	25,100,000
1	24	24	40,400,000
1	48	24	57,000,000
2	1	0	1,520,000
2	4	0	1,390,000
2	12	0	1,810,000
2	24	0	1,810,000
2	48	0	1,810,000
2	1	24	0
2	4	24	0
2	12	24	23,300,000
2	24	24	31,100,000
2	48	24	38,900,000

Solvent codes: 1: 13.3% *w/w* 2: 3.3% *w/w*.
